# Annotations

**Published:** 1903-09-05

**Authors:** 


### ANNOTATIONS.

### Tariffs and Therapeutics.

THE fiscal problem undoubtedly touches many circles, and the controversies it excites are decidedly varied. But it might surely have been expected that it would not ruffle the academic calm of medical science. Yet there is reason to fear that the ferment of disturbance has already commenced to work, and, as an early result, it has involved one of our most respected lay contemporaries in the great gout question. This has already a sufficiently acute aspect. Only within the last month or so we have had a series of essays by rival authorities prefaced by a learned professor's commentary to the effect that silence is best. Now to the consternation of all quiet men it seems that the treatment of gout is an element in the question of tariffs. It is nothing to the point, so we read, to urge that dear bread can be compensated for by cheaper tea and sugar, because sugar is strictly forbidden "to gouty and rheumatic persons." Hence these unfortunate sufferers will be compelled to seek their carbohydrates in the little and expensive loaf. For them, cheap sugar will prove but an additional aggravation. But this is not all. The notion that gout is the special possession of peers and aldermen has, we are told, "long been exploded," for "'poor man's' gout is now a recognised and common variety of the malady." Hence tears are shed for the impecunious sufferer from "poor man's gout" who is consoled for a diminished tale of bread by an invitation not to spare the sugar. We have often to lament our own ignorance, but the omniscience of other people is an even more serious grievance.

### Some Memory Defects.

WITHIN the past few months quite a number of instances have been chronicled by the lay press of complete and more or less sudden loss of the memory of personal identity. Doubtless some of these records rest on a very slender basis of fact, at the same time it is well to remember that there is incontrovertible evidence in favour of the view that the memories of different classes of facts are stored in different areas of the cerebral cortex, and that given the destruction of any one of these areas there will be a corresponding gap in the memory. Thus is to be explained the phenomenon of word-blindness as the result of a lesion disturbing the cortical centres of the left angular gyrus. The power to see the shapes and forms of words remains normal, but as the word-memory is lost the new observation has no significance or meaning. There is reason to believe that the special memories are even more minutely localised in particular areas. For it is possible for a patient to be figure blind without being word-blind; to lose his power of perceiving musical characters whilst retaining his ability to read and understand words; and even to become word-blind without being letter-blind. The inference is that the memories of letters, words, figures, and musical characters are stored in different groups of nerve cells. In the light of such occurrences it is not very difficult to imagine that the memories on which rest the conviction of the continuity of personal identity also have their specific anatomical location, and that pathological changes limited to this may cause the individual to become subjectively destitute of a personal history.

### The Inspection of Workshops.

MRS. GERTRUDE FRANKS has lately been giving in the *Sanitary Record* some of her experiences as a lady workshop inspector in Sheffield under the Factory and Workshops Act of 1901. The duties of the inspectors are to visit the workshops and examine the sanitary arrangements and other details necessary in order that the registers may be properly filled up and a permanent record of the condition of workshops at the time of visiting preserved. To illustrate what such a visit may lead to, Mrs. Franks takes the suppositious case of a recently-opened tailor's workroom. As is frequently the case, the tailor has not notified the factory inspector of the district of his occupation of the workshop which he should have done within one month of occupying it under a penalty not exceeding £5, in order that the workshop might be visited and entered in the register. His plea is ignorance of what was required. Besides this it is noted that he has no abstract of the Factory and Workshops Act affixed, and, seeing that he employs women, he has rendered himself liable to an additional penalty of 40s. It further appears that he is employing too many people for the size of the room, so that the air-space is less than the minimum 250 cubic feet per head. The wretched tailor is then informed that there is not sufficient inlet for fresh air and outlet for foul air, and that he or someone else must provide and maintain proper ventilation, and moreover that the noxious fumes from the gas iron-heater must be removed by means of a hood and shaft carried up into the chimney or through the wall into the open air. Besides these things he must provide an additional w.-c. for the women he employs, must make arrangements to have the walls and ceilings cleaned and whitewashed, and the floor washed once a week, and so forth. We will leave the bewildered tailor here and proceed to moralise. Doubtless there are some who look upon many of the recent enactments, such as the Factory and Workshops Act, which have for their aim the protection of the working classes as merely grandmotherly legislation and instances of modern political degeneracy, but it is probable that most thoughtful people will see in these things a far wider significance and will regard them as evidences of a great spontaneous movement which is now in progress towards bettering the conditions, physical and moral, of the human race, by insisting on what seems scarcely to have been appreciated before in the history of the world, namely the responsibility of the employer of labour and his duty towards the people who work for him. It is to this end that the "spirit of the hive" is moving us now. The cry of survival of the fittest is not heard so loudly in these days as it was a few years ago, and it is more generally appreciated that to strengthen a nation it is not a wise thing to submit the mothers to uncalled-for hardships, for the weakly mother will hardly bring forth a lusty child. On the other hand it is realised that by raising the standard of health among the working classes the employers of labour are themselves among the first to be rewarded.

